# 1-Benzoyl-4-(4-methyl­phen­yl)phthal­azine

**DOI:** 10.1107/S1600536811050641

**Published:** 2011-11-30

**Authors:** Karuppusamy Sakthivel, Kannupal Srinivasan, Sampath Natarajan

**Affiliations:** a School of Chemistry, Bharathidasan University, Thiruchirapalli, Tamil Nadu 620 024, India; b Department of Advanced Technology Fusion, Konkuk University, 1 Hwayang-dong, Gwangjin-gu, Seoul 143 701, Republic of Korea

## Abstract

In the title mol­ecule, C_22_H_16_N_2_O, the tolyl and benzoyl rings make dihedral angles 50.2 (5) and 56.4 (5)°, respectively, with the phthalazine ring system while the dihedral angle between the tolyl and benzoyl rings is 0.70 (4)°. The crystal structure is stabilized by inter­molecular C—H⋯O and C—H⋯N hydrogen bonds, as well as weak C—H⋯π inter­actions.

## Related literature

For the biological activity of phthalazine derivatives, see: Grasso *et al.* (2000 ▶). For related structures, see: Dilek *et al.* (2004 ▶); Rajnikant *et al.* (2006 ▶).
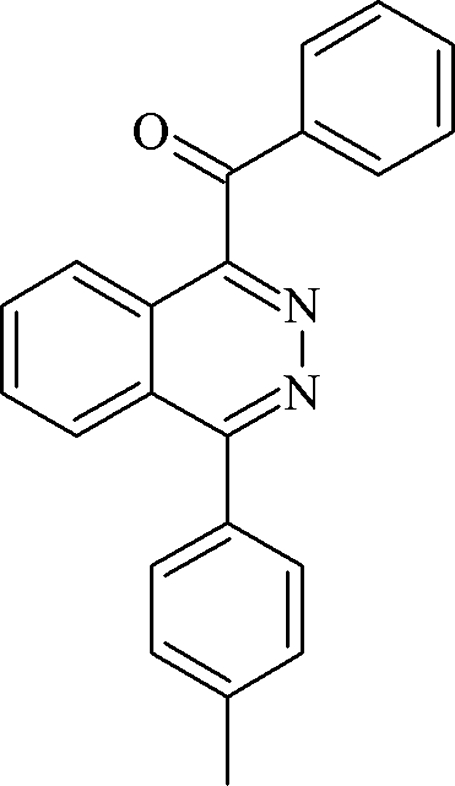


## Experimental

### 

#### Crystal data

C_22_H_16_N_2_O
*M* *_r_* = 324.37
Monoclinic, 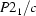

*a* = 12.4873 (2) Å
*b* = 8.8011 (1) Å
*c* = 15.4425 (2) Å
β = 92.458 (1)°
*V* = 1695.60 (4) Å^3^
*Z* = 4
Mo *K*α radiation
μ = 0.08 mm^−1^
*T* = 296 K
0.10 × 0.06 × 0.04 mm

#### Data collection

Bruker SMART APEX CCD area-detector diffractometer
30411 measured reflections
3519 independent reflections
2606 reflections with *I* > 2σ(*I*)
*R*_int_ = 0.036

#### Refinement

*R*[*F* ^2^ > 2σ(*F* ^2^)] = 0.044
*wR*(*F* ^2^) = 0.130
*S* = 1.07
3519 reflections
226 parameters
H-atom parameters constrained
Δρ_max_ = 0.20 e Å^−3^
Δρ_min_ = −0.22 e Å^−3^

### 

Data collection: *APEX2* (Bruker, 2004 ▶); cell refinement: *SAINT* (Bruker, 2004 ▶); data reduction: *SAINT*; program(s) used to solve structure: *SHELXS97* (Sheldrick, 2008 ▶); program(s) used to refine structure: *SHELXL97* (Sheldrick, 2008 ▶); molecular graphics: *ORTEP-3* (Farrugia, 1997 ▶); software used to prepare material for publication: *PLATON* (Spek, 2009 ▶).

## Supplementary Material

Crystal structure: contains datablock(s) I, global. DOI: 10.1107/S1600536811050641/pv2486sup1.cif

Structure factors: contains datablock(s) I. DOI: 10.1107/S1600536811050641/pv2486Isup2.hkl

Supplementary material file. DOI: 10.1107/S1600536811050641/pv2486Isup3.cml

Additional supplementary materials:  crystallographic information; 3D view; checkCIF report

## Figures and Tables

**Table 1:** Hydrogen-bond geometry (Å, °) *Cg*1 is the centroid of the C10–C15 ring.

*D*—H⋯*A*	*D*—H	H⋯*A*	*D*⋯*A*	*D*—H⋯*A*
C12—H12⋯O1^i^	0.93	2.71	3.400 (3)	132
C13—H13⋯N2^ii^	0.93	2.73	3.654 (3)	170
C20—H20⋯N2^iii^	0.93	2.61	3.522 (2)	166
C6—H6⋯*Cg*1^iv^	0.93	2.76	3.549 (2)	143

Symmetry codes: (i) 
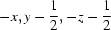
; (ii) 
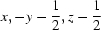
; (iii) 
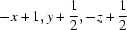
; (iv) 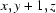.

**Fig. 1.**
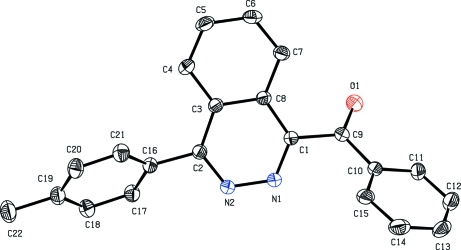
ORTEP diagram of the title molecule with the atom numbering scheme. Displacement ellipsoid are drawn at 30% probability level. H-atoms were removed for clarity.

**Fig. 2.**
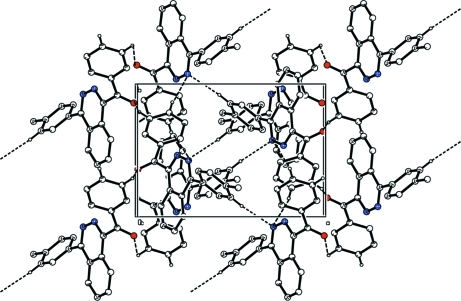
Packing diagram of the title compound viewed down the c axis. Dashed lines indicate the hydrogen bonds between the molecules.

## supplementary crystallographic information

### Comment

Phthalazine (2,3 benzodiazine) is a well known heterocyclic system which is widely used in synthetic organic chemistry as an intermediate. Its derivatives possess remarkable biological activity, such as anticonvulsant antimicrobial, anti-inflammatory, antifungal, antibacterial, vasorelaxant and cardiotonic activity (Grasso *et al.*, 2000).

In the title molecule (Fig. 1), the phthalazine moiety consists of a benzene and a pyridazine rings fused together and shows a planar conformation; the dihedral angle between these rings is 0.70 (4)°. A tolyl and a benzoyl rings are substituted on the pyridazine ring and dihedral angle of these rings with the pyridazine ring are 50.2 (5) and 56.4 (5)°, respectively. Though the molecules do not show any classical hydrogen bonds, these molecules are connected by C–H···O and C—H···N types of intermolecular hydrogen bonds. In addition to these, a C—H···π weak interaction also helps to consolidate the molecules in the unit cell crystal packing (Fig. 2 and Tab. 1). The molecular dimensions in the title compound are in excellent agreement with the corresponding molecular dimensions reported in closely related compounds (Dilek *et al.*, 2004; Rajnikant *et al.*, 2006).

### Experimental

The title compound was synthesized in 70% yield by heating the compounds 1-[2-(4-methylbenzoyl)phenyl]-2-phenylethane-1,2-dione (50 mg, 0.15 mmol) with hydrazine hydrate (11 mg, 0.23 mmol) under reflux in acetonitrile (5 ml) for 6 hr. The crystals suitable for crystallographic study were grown from dichloromethane by slow evaporation at room temperature.

### Refinement

H atoms were positioned geometrically and refined using a riding model with C—H = 0.93 Å for aromatic H and 0.96 Å for methyl H atoms. The *U*_iso_ parameters for H atoms were constraned to be 1.5Ueq of the carrier atom for the methyl H atoms and 1.2Ueq of the carrier atom for the remaining H atoms.

### Crystal data

**Table:**

C_22_H_16_N_2_O	*F*(000) = 680
*M**_r_* = 324.37	*D*_x_ = 1.271 Mg m^−^^3^
Monoclinic, *P*2_1_/*c*	Mo *K*α radiation, λ = 0.71073 Å
Hall symbol: -P 2ybc	Cell parameters from 3519 reflections
*a* = 12.4873 (2) Å	θ = 1.6–26.6°
*b* = 8.8011 (1) Å	µ = 0.08 mm^−^^1^
*c* = 15.4425 (2) Å	*T* = 296 K
β = 92.458 (1)°	Needle, orange
*V* = 1695.60 (4) Å^3^	0.10 × 0.06 × 0.04 mm
*Z* = 4	

### Data collection

**Table:**

Bruker SMART APEX CCD area-detector diffractometer	2606 reflections with *I* > 2σ(*I*)
Radiation source: fine-focus sealed tube	*R*_int_ = 0.036
graphite	θ_max_ = 26.6°, θ_min_ = 1.6°
ω scans	*h* = −15→15
30411 measured reflections	*k* = −11→11
3519 independent reflections	*l* = −19→19

### Refinement

**Table:**

Refinement on *F*^2^	Primary atom site location: structure-invariant direct methods
Least-squares matrix: full	Secondary atom site location: difference Fourier map
*R*[*F*^2^ > 2σ(*F*^2^)] = 0.044	Hydrogen site location: inferred from neighbouring sites
*wR*(*F*^2^) = 0.130	H-atom parameters constrained
*S* = 1.07	*w* = 1/[σ^2^(*F*_o_^2^) + (0.0546*P*)^2^ + 0.4182*P*] where *P* = (*F*_o_^2^ + 2*F*_c_^2^)/3
3519 reflections	(Δ/σ)_max_ = 0.003
226 parameters	Δρ_max_ = 0.20 e Å^−^^3^
0 restraints	Δρ_min_ = −0.22 e Å^−^^3^

### Special details

**Table:**

Geometry. All e.s.d.'s (except the e.s.d. in the dihedral angle between two l.s. planes) are estimated using the full covariance matrix. The cell e.s.d.'s are taken into account individually in the estimation of e.s.d.'s in distances, angles and torsion angles; correlations between e.s.d.'s in cell parameters are only used when they are defined by crystal symmetry. An approximate (isotropic) treatment of cell e.s.d.'s is used for estimating e.s.d.'s involving l.s. planes.
Refinement. Refinement of *F*^2^ against ALL reflections. The weighted *R*-factor *wR* and goodness of fit *S* are based on *F*^2^, conventional *R*-factors *R* are based on *F*, with *F* set to zero for negative *F*^2^. The threshold expression of *F*^2^ > σ(*F*^2^) is used only for calculating *R*-factors(gt) *etc*. and is not relevant to the choice of reflections for refinement. *R*-factors based on *F*^2^ are statistically about twice as large as those based on *F*, and *R*- factors based on ALL data will be even larger.

### Fractional atomic coordinates and isotropic or equivalent isotropic displacement parameters (Å^2^)

**Table:**

	*x*	*y*	*z*	*U*_iso_*/*U*_eq_	
O1	0.00887 (10)	0.14263 (15)	−0.07710 (9)	0.0706 (4)	
N1	0.21706 (11)	0.03719 (15)	0.05410 (9)	0.0506 (3)	
N2	0.27574 (11)	0.07622 (15)	0.12807 (9)	0.0503 (3)	
C8	0.17846 (12)	0.30203 (17)	0.02488 (10)	0.0429 (3)	
C1	0.17027 (12)	0.14321 (17)	0.00642 (10)	0.0447 (4)	
C2	0.28673 (12)	0.22046 (18)	0.15030 (10)	0.0449 (4)	
C3	0.23980 (12)	0.34210 (17)	0.10025 (10)	0.0450 (4)	
C4	0.24861 (16)	0.49746 (19)	0.12311 (12)	0.0611 (5)	
H4	0.2868	0.5256	0.1736	0.073*	
C5	0.20122 (17)	0.6061 (2)	0.07141 (13)	0.0660 (5)	
H5	0.2077	0.7079	0.0868	0.079*	
C6	0.14337 (15)	0.56618 (19)	−0.00404 (12)	0.0607 (5)	
H6	0.1127	0.6418	−0.0391	0.073*	
C7	0.13101 (13)	0.41727 (19)	−0.02740 (11)	0.0521 (4)	
H7	0.0914	0.3919	−0.0777	0.063*	
C9	0.09674 (14)	0.08398 (18)	−0.06611 (10)	0.0503 (4)	
C10	0.12936 (15)	−0.04512 (18)	−0.12021 (10)	0.0540 (4)	
C11	0.04861 (19)	−0.1217 (2)	−0.16781 (12)	0.0716 (6)	
H11	−0.0224	−0.0922	−0.1631	0.086*	
C12	0.0726 (3)	−0.2391 (3)	−0.22112 (14)	0.0957 (8)	
H12	0.0183	−0.2896	−0.2525	0.115*	
C13	0.1773 (3)	−0.2824 (3)	−0.22833 (14)	0.1016 (10)	
H13	0.1936	−0.3623	−0.2649	0.122*	
C14	0.2591 (2)	−0.2087 (3)	−0.18174 (14)	0.0890 (8)	
H14	0.3298	−0.2393	−0.1870	0.107*	
C15	0.23530 (17)	−0.0885 (2)	−0.12707 (11)	0.0643 (5)	
H15	0.2897	−0.0382	−0.0957	0.077*	
C16	0.35062 (12)	0.24271 (18)	0.23300 (10)	0.0478 (4)	
C17	0.32650 (14)	0.1561 (2)	0.30488 (11)	0.0541 (4)	
H17	0.2690	0.0890	0.3010	0.065*	
C18	0.38613 (14)	0.1678 (2)	0.38175 (11)	0.0596 (5)	
H18	0.3686	0.1079	0.4287	0.071*	
C19	0.47198 (14)	0.2676 (2)	0.39033 (12)	0.0605 (5)	
C20	0.49709 (15)	0.3524 (2)	0.31882 (13)	0.0658 (5)	
H20	0.5553	0.4183	0.3227	0.079*	
C21	0.43727 (14)	0.3414 (2)	0.24106 (12)	0.0606 (5)	
H21	0.4554	0.4007	0.1940	0.073*	
C22	0.53529 (18)	0.2818 (3)	0.47533 (14)	0.0875 (7)	
H22A	0.5060	0.2145	0.5171	0.131*	
H22B	0.5314	0.3846	0.4958	0.131*	
H22C	0.6088	0.2555	0.4671	0.131*	

### Atomic displacement parameters (Å^2^)

**Table:**

	*U*^11^	*U*^22^	*U*^33^	*U*^12^	*U*^13^	*U*^23^
O1	0.0648 (8)	0.0652 (8)	0.0797 (9)	0.0137 (7)	−0.0217 (7)	−0.0093 (7)
N1	0.0605 (8)	0.0396 (7)	0.0507 (8)	0.0036 (6)	−0.0103 (6)	−0.0013 (6)
N2	0.0585 (8)	0.0414 (7)	0.0500 (8)	0.0032 (6)	−0.0097 (6)	0.0009 (6)
C8	0.0470 (8)	0.0402 (8)	0.0417 (8)	0.0035 (6)	0.0054 (6)	0.0015 (6)
C1	0.0503 (9)	0.0402 (8)	0.0433 (8)	0.0041 (7)	−0.0009 (7)	−0.0005 (6)
C2	0.0481 (8)	0.0434 (8)	0.0433 (8)	−0.0008 (7)	0.0023 (6)	−0.0004 (6)
C3	0.0521 (9)	0.0400 (8)	0.0431 (8)	0.0014 (7)	0.0043 (7)	−0.0006 (6)
C4	0.0808 (12)	0.0418 (9)	0.0604 (11)	0.0007 (8)	−0.0029 (9)	−0.0081 (8)
C5	0.0881 (13)	0.0362 (9)	0.0740 (12)	0.0027 (9)	0.0056 (10)	−0.0027 (8)
C6	0.0724 (11)	0.0434 (9)	0.0666 (11)	0.0116 (8)	0.0078 (9)	0.0124 (8)
C7	0.0581 (9)	0.0485 (9)	0.0497 (9)	0.0075 (8)	0.0015 (7)	0.0061 (7)
C9	0.0608 (10)	0.0430 (8)	0.0463 (9)	0.0040 (7)	−0.0073 (7)	0.0026 (7)
C10	0.0795 (12)	0.0403 (8)	0.0413 (8)	0.0036 (8)	−0.0072 (8)	0.0037 (7)
C11	0.1067 (16)	0.0528 (10)	0.0531 (10)	−0.0058 (10)	−0.0220 (10)	−0.0002 (8)
C12	0.169 (3)	0.0557 (13)	0.0598 (13)	0.0008 (15)	−0.0239 (15)	−0.0070 (10)
C13	0.211 (3)	0.0494 (12)	0.0442 (11)	0.0230 (17)	0.0024 (16)	−0.0069 (9)
C14	0.141 (2)	0.0679 (13)	0.0594 (12)	0.0400 (14)	0.0193 (13)	0.0068 (11)
C15	0.0881 (13)	0.0544 (10)	0.0504 (10)	0.0150 (10)	0.0040 (9)	0.0048 (8)
C16	0.0494 (9)	0.0478 (9)	0.0460 (9)	0.0001 (7)	−0.0004 (7)	−0.0040 (7)
C17	0.0560 (10)	0.0568 (10)	0.0495 (9)	−0.0071 (8)	−0.0001 (7)	0.0006 (8)
C18	0.0622 (11)	0.0684 (12)	0.0479 (9)	−0.0002 (9)	−0.0001 (8)	0.0006 (8)
C19	0.0551 (10)	0.0716 (12)	0.0541 (10)	0.0070 (9)	−0.0069 (8)	−0.0126 (9)
C20	0.0542 (10)	0.0733 (13)	0.0690 (12)	−0.0138 (9)	−0.0054 (9)	−0.0104 (10)
C21	0.0603 (10)	0.0619 (11)	0.0596 (11)	−0.0127 (8)	0.0030 (8)	0.0014 (9)
C22	0.0823 (15)	0.1086 (18)	0.0694 (14)	0.0066 (13)	−0.0233 (11)	−0.0172 (13)

### Geometric parameters (Å, °)

**Table:**

O1—C9	1.2180 (19)	C11—H11	0.9300
N1—C1	1.3105 (19)	C12—C13	1.371 (4)
N1—N2	1.3740 (17)	C12—H12	0.9300
N2—C2	1.321 (2)	C13—C14	1.385 (4)
C8—C3	1.410 (2)	C13—H13	0.9300
C8—C7	1.411 (2)	C14—C15	1.393 (3)
C8—C1	1.429 (2)	C14—H14	0.9300
C1—C9	1.510 (2)	C15—H15	0.9300
C2—C3	1.431 (2)	C16—C21	1.389 (2)
C2—C16	1.489 (2)	C16—C17	1.390 (2)
C3—C4	1.415 (2)	C17—C18	1.378 (2)
C4—C5	1.364 (3)	C17—H17	0.9300
C4—H4	0.9300	C18—C19	1.388 (3)
C5—C6	1.389 (3)	C18—H18	0.9300
C5—H5	0.9300	C19—C20	1.380 (3)
C6—C7	1.366 (2)	C19—C22	1.508 (2)
C6—H6	0.9300	C20—C21	1.390 (2)
C7—H7	0.9300	C20—H20	0.9300
C9—C10	1.478 (2)	C21—H21	0.9300
C10—C15	1.385 (3)	C22—H22A	0.9600
C10—C11	1.396 (3)	C22—H22B	0.9600
C11—C12	1.363 (3)	C22—H22C	0.9600
			
C1—N1—N2	119.87 (13)	C11—C12—H12	120.1
C2—N2—N1	120.15 (13)	C13—C12—H12	120.1
C3—C8—C7	119.50 (14)	C12—C13—C14	120.8 (2)
C3—C8—C1	116.16 (13)	C12—C13—H13	119.6
C7—C8—C1	124.34 (15)	C14—C13—H13	119.6
N1—C1—C8	123.91 (14)	C13—C14—C15	119.9 (2)
N1—C1—C9	114.40 (13)	C13—C14—H14	120.0
C8—C1—C9	121.43 (13)	C15—C14—H14	120.0
N2—C2—C3	122.89 (14)	C10—C15—C14	119.0 (2)
N2—C2—C16	113.28 (13)	C10—C15—H15	120.5
C3—C2—C16	123.82 (14)	C14—C15—H15	120.5
C8—C3—C4	118.76 (15)	C21—C16—C17	117.78 (15)
C8—C3—C2	117.00 (14)	C21—C16—C2	123.07 (15)
C4—C3—C2	124.21 (15)	C17—C16—C2	119.08 (14)
C5—C4—C3	120.25 (17)	C18—C17—C16	121.29 (16)
C5—C4—H4	119.9	C18—C17—H17	119.4
C3—C4—H4	119.9	C16—C17—H17	119.4
C4—C5—C6	120.71 (16)	C17—C18—C19	121.09 (17)
C4—C5—H5	119.6	C17—C18—H18	119.5
C6—C5—H5	119.6	C19—C18—H18	119.5
C7—C6—C5	120.83 (16)	C20—C19—C18	117.83 (16)
C7—C6—H6	119.6	C20—C19—C22	121.52 (19)
C5—C6—H6	119.6	C18—C19—C22	120.65 (19)
C6—C7—C8	119.90 (16)	C19—C20—C21	121.44 (17)
C6—C7—H7	120.0	C19—C20—H20	119.3
C8—C7—H7	120.0	C21—C20—H20	119.3
O1—C9—C10	121.04 (15)	C16—C21—C20	120.56 (17)
O1—C9—C1	118.19 (15)	C16—C21—H21	119.7
C10—C9—C1	120.74 (14)	C20—C21—H21	119.7
C15—C10—C11	119.84 (18)	C19—C22—H22A	109.5
C15—C10—C9	122.84 (16)	C19—C22—H22B	109.5
C11—C10—C9	117.29 (17)	H22A—C22—H22B	109.5
C12—C11—C10	120.8 (2)	C19—C22—H22C	109.5
C12—C11—H11	119.6	H22A—C22—H22C	109.5
C10—C11—H11	119.6	H22B—C22—H22C	109.5
C11—C12—C13	119.7 (2)		
			
C1—N1—N2—C2	−1.3 (2)	C8—C1—C9—C10	142.14 (16)
N2—N1—C1—C8	1.9 (2)	O1—C9—C10—C15	161.87 (17)
N2—N1—C1—C9	−172.33 (14)	C1—C9—C10—C15	−20.2 (2)
C3—C8—C1—N1	−1.1 (2)	O1—C9—C10—C11	−16.1 (2)
C7—C8—C1—N1	178.05 (15)	C1—C9—C10—C11	161.76 (15)
C3—C8—C1—C9	172.76 (14)	C15—C10—C11—C12	0.0 (3)
C7—C8—C1—C9	−8.1 (2)	C9—C10—C11—C12	178.10 (17)
N1—N2—C2—C3	−0.1 (2)	C10—C11—C12—C13	−0.2 (3)
N1—N2—C2—C16	178.77 (13)	C11—C12—C13—C14	0.3 (4)
C7—C8—C3—C4	2.4 (2)	C12—C13—C14—C15	−0.3 (3)
C1—C8—C3—C4	−178.43 (15)	C11—C10—C15—C14	0.0 (3)
C7—C8—C3—C2	−179.47 (14)	C9—C10—C15—C14	−177.99 (16)
C1—C8—C3—C2	−0.3 (2)	C13—C14—C15—C10	0.2 (3)
N2—C2—C3—C8	0.9 (2)	N2—C2—C16—C21	128.64 (18)
C16—C2—C3—C8	−177.89 (14)	C3—C2—C16—C21	−52.5 (2)
N2—C2—C3—C4	178.88 (16)	N2—C2—C16—C17	−48.2 (2)
C16—C2—C3—C4	0.1 (3)	C3—C2—C16—C17	130.66 (17)
C8—C3—C4—C5	−2.0 (3)	C21—C16—C17—C18	0.2 (3)
C2—C3—C4—C5	179.96 (17)	C2—C16—C17—C18	177.20 (16)
C3—C4—C5—C6	0.3 (3)	C16—C17—C18—C19	0.5 (3)
C4—C5—C6—C7	1.2 (3)	C17—C18—C19—C20	−1.3 (3)
C5—C6—C7—C8	−0.8 (3)	C17—C18—C19—C22	178.72 (18)
C3—C8—C7—C6	−1.0 (2)	C18—C19—C20—C21	1.4 (3)
C1—C8—C7—C6	179.88 (16)	C22—C19—C20—C21	−178.63 (19)
N1—C1—C9—O1	134.47 (17)	C17—C16—C21—C20	−0.1 (3)
C8—C1—C9—O1	−39.9 (2)	C2—C16—C21—C20	−176.99 (16)
N1—C1—C9—C10	−43.5 (2)	C19—C20—C21—C16	−0.7 (3)

### Hydrogen-bond geometry (Å, °)

**Table:**

Cg1 is the centroid of the C10–C15 ring.

**Table:**

*D*—H···*A*	*D*—H	H···*A*	*D*···*A*	*D*—H···*A*
C12—H12···O1^i^	0.93	2.71	3.400 (3)	132
C13—H13···N2^ii^	0.93	2.73	3.654 (3)	170
C20—H20···N2^iii^	0.93	2.61	3.522 (2)	166
C6—H6···Cg1^iv^	0.93	2.76	3.549 (2)	143

Symmetry codes: (i) −*x*, *y*−1/2, −*z*−1/2; (ii) *x*, −*y*−1/2, *z*−1/2; (iii) −*x*+1, *y*+1/2, −*z*+1/2; (iv) *x*, *y*+1, *z*.
